# Current barriers to initiating insulin therapy in individuals with type 2 diabetes

**DOI:** 10.3389/fendo.2024.1366368

**Published:** 2024-03-14

**Authors:** Alba Galdón Sanz-Pastor, Alicia Justel Enríquez, Ana Sánchez Bao, Francisco Javier Ampudia-Blasco

**Affiliations:** ^1^ Department of Endocrinology and Nutrition, Gregorio Marañón General University Hospital, Madrid, Spain; ^2^ Department of Medicine, Complutense University of Madrid, Madrid, Spain; ^3^ Department of Endocrinology and Nutrition, La Princesa University Hospital, Madrid, Spain; ^4^ Department of Endocrinology and Nutrition, Ferrol University Hospital Complex, Ferrol, A Coruña, Spain; ^5^ Department of Medicine, Medicine Faculty, University of Valencia (UV), Valencia, Spain; ^6^ Department of Endocrinology & Nutrition, Clinic University Hospital of Valencia, Valencia, Spain; ^7^ INCLIVA Biomedical Research Institute, Valencia, Spain; ^8^ Biomedical Research Networking Center for Diabetes and Associated Metabolic Diseases (CIBERDEM), Biomedical Research Networking Center (CIBER) of Diabetes and Associated Metabolic Diseases, Madrid, Spain

**Keywords:** type 2 diabetes, insulinization, glycemic control, therapeutic inertia, hypoglycemia, adherence, once weekly insulin

## Abstract

Insulin is an essential drug in the treatment of diabetes, often necessary for managing hyperglycemia in type 2 diabetes mellitus (T2DM). It should be considered in cases of severe hyperglycemia requiring hospitalization, after the failure of other treatments, in advanced chronic kidney disease, liver cirrhosis, post-transplant diabetes, or during pregnancy. Moreover, in specific patient subgroups, early initiation of insulin is crucial for hyperglycemia control and prevention of chronic complications. Clinical guidelines recommend initiating insulin when other treatments fail, although there are barriers that may delay its initiation. The timing of initiation depends on individual patient characteristics. Typically, insulinization starts by adding basal insulin to the patient’s existing treatment and, if necessary, progresses by gradually introducing prandial insulin. Several barriers have been identified that hinder the initiation of insulin, including fear of hypoglycemia, lack of adherence, the need for glucose monitoring, the injection method of insulin administration, social rejection associated with the stigma of injections, weight gain, a sense of therapeutic failure at initiation, lack of experience among some healthcare professionals, and the delayed and reactive positioning of insulin in recent clinical guidelines. These barriers contribute, among other factors, to therapeutic inertia in initiating and intensifying insulin treatment and to patients’ non-adherence. In this context, the development of once-weekly insulin formulations could improve initial acceptance, adherence, treatment satisfaction, and consequently, the quality of life for patients. Currently, two once-weekly basal insulins, insulin icodec and basal insulin BIF, which are in different stages of clinical development, may help. Their longer half-life translates to lower variability and reduced risk of hypoglycemia. This review addresses the need for insulin in T2DM, its positioning in clinical guidelines under specific circumstances, the current barriers to initiating and intensifying insulin treatment, and the potential role of once-weekly insulin formulations as a potential solution to facilitate timely initiation of insulinization, which would reduce therapeutic inertia and achieve better early control in people with T2DM.

## Introduction

**Figure 1:**
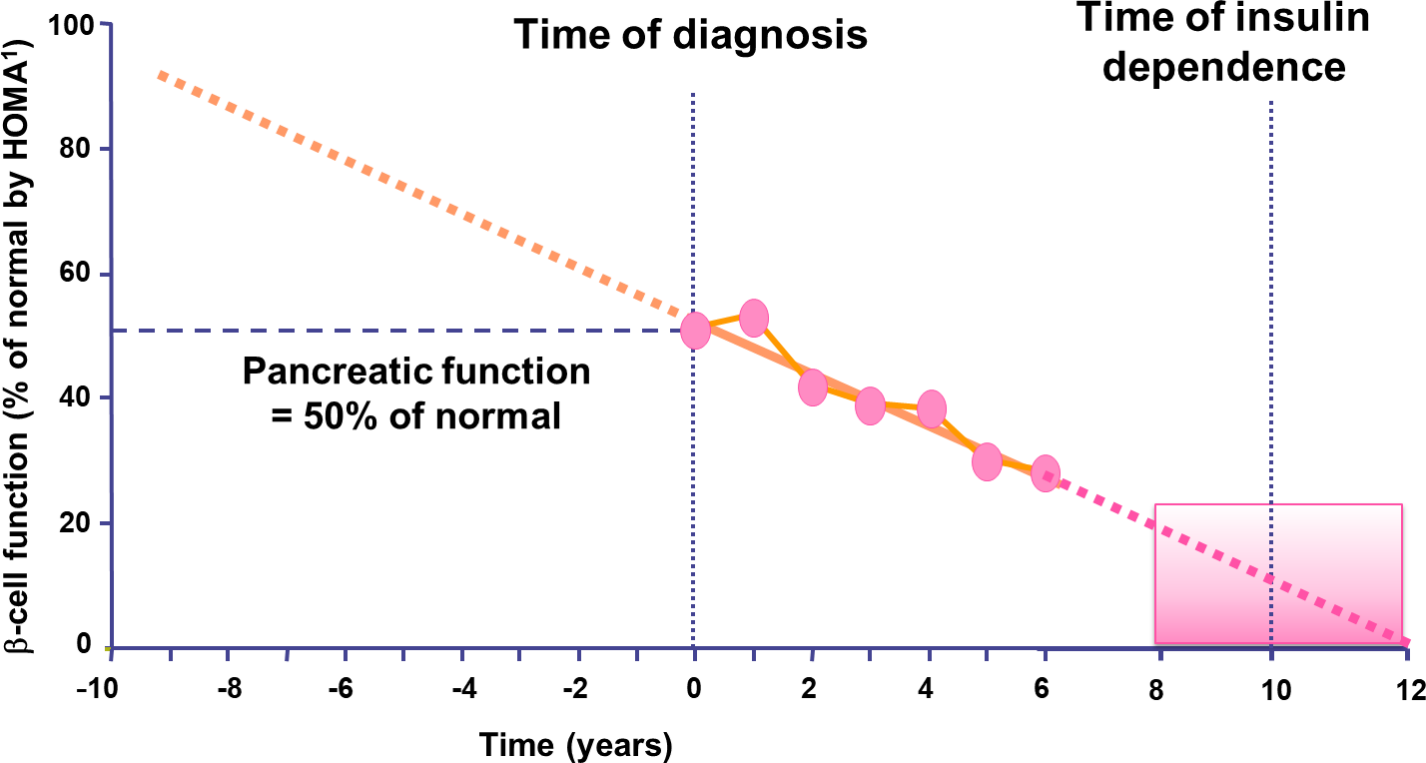
Type 2 diabetes is progressive and intensification of insulin treatment will be needed over time. Extrapolation of decline in β-cell function suggests that deterioration in β-cell function may commence 10–12 years before diabetes diagnosis. Adapted from (1). ^1^ Homeostasis Model Assessment.

Insulin is necessary in type 2 diabetes mellitus (T2DM). Since the UK Prospective Diabetes Study (UKPDS), it has been known that the progressive deterioration of insulin secretion over time leads to the failure of non-insulin therapies, necessitating insulin intensification in patients with T2DM (1) (**Figure 1**). Insulin treatment in T2DM is well-established and considered a safe and effective therapy, particularly in specific clinical situations. More recently, certain patient subgroups, notably those with severe insulin deficiency (SIDD), have been identified that potentially require early insulinization, since they are associated with poor glycemic control and a higher risk of microvascular complications (2).

Current recommendations advise initiating insulin when nutritional therapy and physical exercise, along with other non-insulin medications, fail to achieve control objectives (3–5). However, these therapeutic guidelines identify specific clinical situations where insulinization is a preferred option. It is now acknowledged that insulinization should begin with the addition of basal insulin to the patient’s existing treatment and, if necessary, progress by gradually introducing prandial insulin. However, there are various barriers to insulin therapy, on the part of both patients and healthcare professionals, which unjustifiably delay the initiation of insulinization or influence patients’ non-adherence to insulin treatment (6). Undoubtedly, the daily administration of one or more doses of insulin represents one of the most significant barriers to insulin treatment.

This review analyzes various aspects related to insulin treatment in T2DM, with a special emphasis on current barriers to the initiation of insulinization. Additionally, potential solutions are introduced to reduce barriers to timely insulin initiation and thereby contribute to achieving and maintaining control objectives in T2DM.

## Why is insulin necessary for people with type 2 diabetes?

The main pathophysiological defects underlying the onset and progression of T2DM are pancreatic β-cell insufficiency and insulin resistance, primarily in skeletal muscle, liver, and adipose tissue. The release and action of insulin must precisely meet metabolic demands. Therefore, both the molecular mechanisms involved in insulin synthesis and secretion, as well as tissue-level responses, are crucial for adequate glycemic control. Additionally, other defects in multiple organs that may contribute to the development of T2DM have been identified. This perspective was postulated by DeFronzo in 2009 and is known as the ominous octet (7).

**Figure 2:**
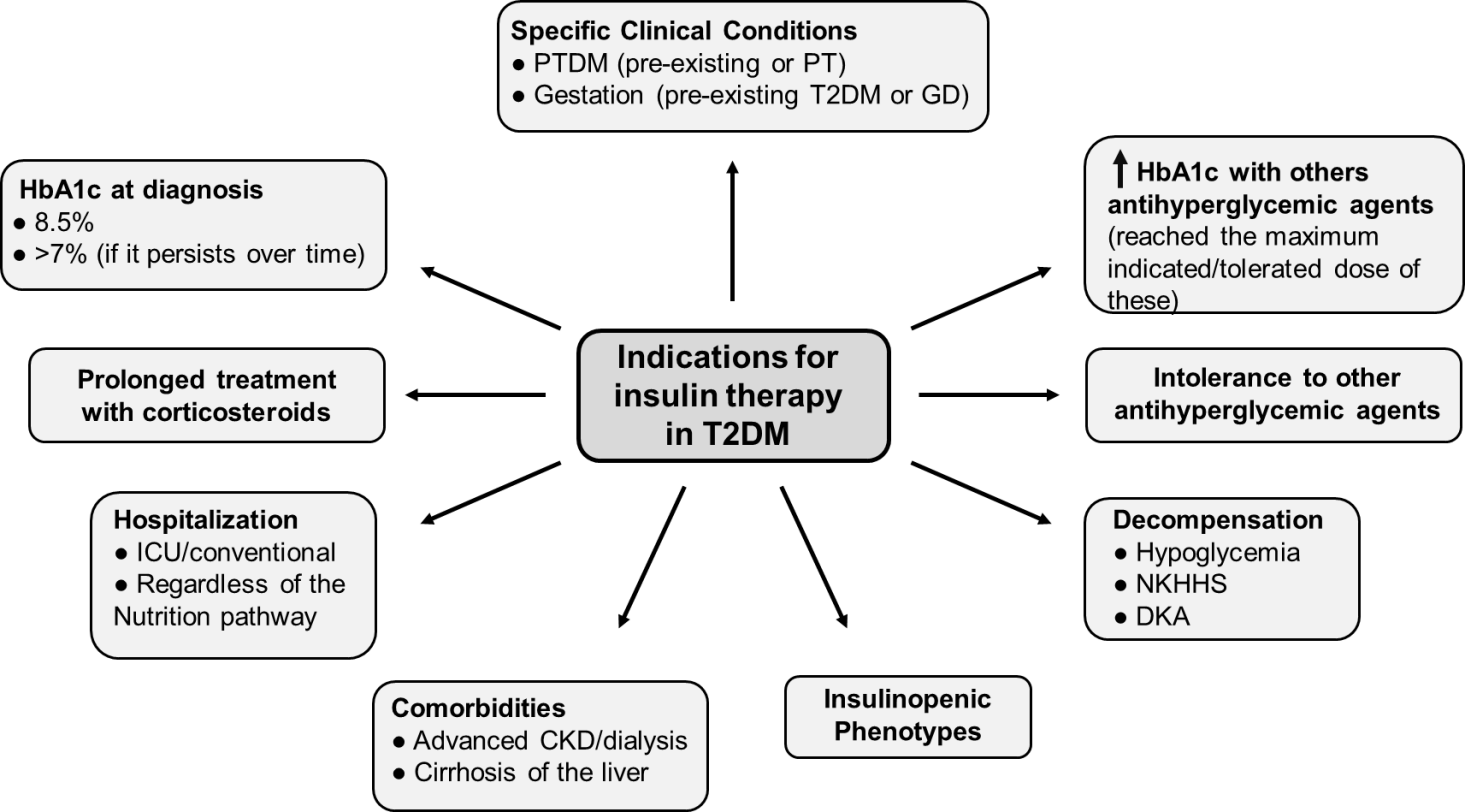
People with type 2 diabetes who are candidates for insulin therapy Modified from (9). Situations in which the patient’s characteristics or clinical condition, make them candidates for the use of basal insulin are described. CKD, chronic kidney disease; DKA, diabetic ketoacidosis; GD, gestational diabetes; HbA1c, glycosylated hemoglobin; ICU, Intensive Care Unit; NKHHS, nonketotic hyperosmolar hyperglycemic state; PT, post-transplant; PTDM, post-transplant diabetes mellitus; T2DM, type 2 diabetes mellitus.

Not all individuals with T2DM will require insulin treatment. In many cases, good metabolic control can be achieved through lifestyle changes, nutritional therapy, and non-insulin hypoglycemic agents. However, despite the numerous pharmacological options available today, insulin remains a necessary, effective, and safe treatment, particularly in specific clinical situations, as described below (8, 9) (**Figure 2**).

### Severe hyperglycemia at the time of presentation

Insulin may be the initial treatment for some patients with T2DM, depending on the severity of the metabolic disturbance at its onset. In cases of symptomatic hyperglycemia with weight loss, polydipsia, polyuria, or severe hyperglycemia with ketonuria, insulin is the preferred initial treatment. In T2DM, pancreatic β-cell insufficiency has been associated with a loss of 24-65% of β-cell mass and a loss of 50-97% of their secretory capacity. Initially, hyperinsulinemia manages to overcome insulin resistance in peripheral tissues. However, if this situation persists, it leads to an insufficiency in the secretory capacity of β-cells, resulting in marked hyperglycemia. Associated mechanisms include insulin resistance, glucotoxicity, lipotoxicity, β-cell senescence, and apoptosis (10). Additionally, it has been suggested that intensive insulin therapy in the first weeks or months after the diagnosis of severe hyperglycemia could, in addition to improving hyperglycemia, reverse damage caused by glucotoxicity and reduce the metabolic memory footprint (7).

### Poor glycemic control despite other medications

Achieving glycemic control goals as soon as possible is crucial to avoid chronic complications and associated mortality in T2DM (11). However, according to data from the Centers for Disease Control and Prevention in the US, a high proportion of patients are still far from achieving adequate metabolic control (8). Traditional recommendations for T2DM treatment have been based on sequential, progressive intensification of medications as metabolic control deteriorates over time. However, there is therapeutic inertia in clinical practice, and often there is a delay in treatment intensification (12).

Regarding insulin, there is no general agreement on how to avoid therapeutic inertia in insulin prescription when it is genuinely needed, as reflected in the wide variety of recommendations in clinical practice guidelines (13). However, due to the progressive nature of T2DM, many patients would benefit from earlier insulinization, allowing them to achieve and maintain the individualized control goal for each patient more promptly (8). It is essential to explain to patients the progressive nature of T2DM objectively. Insulin should not be portrayed as a threat or described as a sign of failure or personal punishment. It is imperative to dispel any notion that insulin therapy serves as a punitive measure consequent to non-compliance with recommended lifestyle interventions. On the contrary, emphasis should be placed on the utility and importance of insulin in maintaining glycemic control in case of disease progression when the effect of other non-insulin hypoglycemic agents is insufficient (14).

### Acute clinical situations

T2DM is a risk factor for hospitalization. Hyperglycemia in the hospital is associated with increased complications during hospitalization (infections, prolonged hospital stay, poor wound healing) and in-hospital mortality (15, 16). The safety and efficacy of non-insulin therapies in the hospital setting are an active area of research (17). However, for glycemic control in intensive care units, intravenous insulin administration remains the treatment of choice. Intravenous insulin administration allows greater dosing flexibility by adjusting the infusion rate to adapt to fluctuations in the clinical situation and insulin doses needed. In conventional hospital wards outside of intensive care units, insulin remains the treatment of choice for patients with T2DM. For patients with erratic oral intake, basal insulin with or without correction boluses is the preferred treatment. In those patients with adequate nutritional intake, the basal-bolus regimen with correction components is also the treatment of choice for most patients. Finally, in patients with enteral or parenteral nutrition to correct or prevent hyperglycemia, the use of subcutaneous rapid-acting insulin every 4 or 6 hours could be considered (15–17).

### Special clinical situations

#### Advanced chronic kidney disease

In patients undergoing hemodialysis, the utilization of non-insulin antihyperglycemic medications is notably limited, with insulin remaining the preferred treatment option (18). However, the prematurely halted Flow study, which demonstrated renal benefits with semaglutide, may change this recommendation (19). For individuals with advanced chronic kidney disease (CKD), particularly those not undergoing hemodialysis, guidelines such as KDIGO prioritize the use of glucagon-like peptide-1 receptor agonists (GLP-1 RAs). These agents exhibit sustained hypoglycemic effects even at filtration rates as low as 15 mL/min/1.73 m^2^ and offer additional benefits not observed with insulin (20). Consequently, in such patients, if insulin initiation becomes necessary, discontinuation of these medications should be avoided if gastrointestinal tolerance remains acceptable, owing to the supplementary positive effects they confer.

#### Liver cirrhosis

In patients with advanced liver disease, the selection of non-insulin antihyperglycemic drugs is complex. A high proportion of patients have concomitant malnutrition. Additionally, most non-insulin antihyperglycemic drugs are metabolized in the liver. In the case of liver failure, the administration of these drugs is associated with greater adverse effects due to abnormally elevated concentrations. In individuals with T2DM and liver disease classified as Child-Pugh Class B or C, the use of non-insulin antihyperglycemic medications should be approached with caution or altogether avoided. Insulin remains the primary treatment for managing hyperglycemia in this patient population (21).

#### Post-transplantation

In patients with pre-existing T2DM and those who develop post-transplant diabetes mellitus (PTDM), sustained hyperglycemia significantly increases morbidity and mortality. The presence of frequent comorbidities and the use of immunosuppressants influence treatment choice. The most recent consensus recommends the use of insulin for treating hyperglycemia post-transplantation surgery. However, for stable patients, oral or non-insulin injectable agents (either alone or in combination) may be preferred, unless optimal diabetes control cannot be attained (22).

#### Pregnancy

Nutritional treatment and adapted physical exercise are the initial treatment for women with gestational diabetes (GD) and those with pregestational T2DM. However, when these measures are insufficient to achieve and maintain adequate glycemic control, insulin is the drug of choice to treat hyperglycemia during pregnancy (23).

### Personalized medicine for different phenotypes of patients with T2DM

Several studies have identified phenotypes or clusters of patients with T2DM characterized by severe insulin deficiency. The insulinopenic phenotype shares similarities with the autoimmune diabetes phenotype (younger age, lower body mass index [BMI], poorer metabolic control, and greater glycemic variability) but does not show autoantibodies. This group of patients often has higher levels of glycated hemoglobin (HbA1c), a high incidence of ketoacidosis, early development of diabetic retinopathy, a high prevalence of diabetic neuropathy, and a greater risk of macrovascular complications and diabetic kidney disease (24).

In studies conducted in the Asian population, a higher prevalence of patients with T2DM with severe insulinopenia has been observed, with insulin treatment in early stages, lower β-cell function, lower insulin resistance, and lower BMI, especially among the Indian and Chinese populations compared to the European population. This patient cohort exhibits a more aggressive disease progression and a higher risk of complications (25).

While we do not have prospective studies exploring the initial choice of pharmacological treatment based on different groups, the proposed new phenotypes of T2DM provide new perspectives for personalized treatments based on a better understanding of the pathophysiology (10). Based on the described findings, it is expected that in certain patient subgroups, earlier initiation of insulin treatment may be crucial to improving control and preventing acute and chronic complications associated with diabetes.

## When and how to initiate insulin therapy in people with type 2 diabetes? current recommendations according to different clinical guidelines

The consensus report from the American Diabetes Association (ADA) and the European Association for the Study of Diabetes (EASD), titled “Management of Hyperglycemia in Type 2 Diabetes”, recommends a holistic, multifactorial, and patient-centered approach to diabetes care. Specific factors influencing treatment choice include individualized glycemic and weight goals, cardio-renal protection, underlying physiological factors, side effects, access, cost, and availability (3). The progressive nature of T2DM often necessitates the initiation of insulin therapy. This scenario should be explained to patients, avoiding the use of insulin as a threat or portraying it as a sign of failure. Instead, emphasis should be placed on the utility and importance of insulin in maintaining glycemic control once the disease progression renders other medications less effective. Currently, basal insulin is considered the preferred regimen following the failure of non-insulin antidiabetic drugs (3).

Determining the appropriate timing for insulin therapy initiation in people with T2DM is challenging. Traditionally, many clinical practice guidelines recommended introducing insulin based on a specific HbA1c value. However, recent consensus statements for hyperglycemia treatment in T2DM by the ADA and EASD (3) make it clear that the HbA1c target for each patient should be individualized based on multiple parameters, including the risk of hypoglycemia. This patient-centered, multifactorial approach means that a single HbA1c value cannot be considered an adequate indicator of the need to start insulin therapy. The timing for initiating this therapy will depend on each patient’s characteristics, including age, duration of diabetes, weight, risk of hypoglycemia, etc. (3).

Clinical practice guidelines establish criteria to help decide when to initiate insulin therapy (**Table 1**). According to the American Association of Clinical Endocrinology (AACE), insulinization should be initiated when therapy, including lifestyle modifications and non-insulin medications, fails to achieve glycemic control goals, or whenever a patient, whether previously exposed to insulin or not, presents symptoms of hyperglycemia. Specifically, AACE indicates that patients with HbA1c >10%, symptomatic with polyuria, polydipsia, or polyphagia, will benefit more from starting insulin (4). However, if they have few symptoms or no significant symptoms, these patients can initiate therapy with maximum doses of two or three non-insulin medications (4). This guideline also recommends using insulin in patients who need to intensify glycemic control and are already receiving three to four non-insulin medications (27). The ADA/EASD positioning in 2023 considers that in patients with insufficient glycemic control despite the use of three non-insulin antidiabetic drugs in combination, insulinization should be considered. It also contemplates early introduction of insulin if there is evidence of acute decompensation with weight loss, ketonuria/ketosis, or other symptoms of hyperglycemia, or when HbA1c levels are >10% or blood glucose levels are ≥300 mg/dL. In some cases, as glucotoxicity resolves, it may be possible to simplify the insulin regimen and/or suspend insulin and reintroduce a combination of non-insulin medications.

**Table 1:** Recommendations in the current clinical guidelines regarding the use of insulin in people with T2DM.

Clinical Guidelines	When to Start with Insulin	How to Start with Insulin
ADA/ EASD 2024 (3)	- Blood glucose >300 mg/dL, HbA1c >10%, symptoms of hyperglycemia, or evidence of catabolism. - Patients on treatment with 3 non-insulin therapies and insufficient control.	Start with basal insulin 0.1-0.2 U/kg/day with individualized titration for days or weeks.
		Intensify with prandial insulin 4 U or 10% of basal insulin at the largest meal or the meal with the greatest postprandial excursion. Intensify according to individual needs
AACE 2023 (4)	- If symptomatic hyperglycemia, HbA1c >10% and/or blood glucose >300 mg/dL (signs of marked insulin deficiency). - Patients on treatment with 3-4 non-insulin therapies that do not reach glycemic targets.	Start with basal insulin 0.1 to 0.2 U/kg/day if HbA1c <8% or 0.2 to 0.3 U/kg/day if HbA1c >8%. Adjust every 2-3 days for FCG <110 mg/dL without hypoglycemia.
		Intensify with prandial insulin: Start a dose at the largest meal (10% of the basal dose or 5 U). Add to other meals as needed. or Start at all meals at 50% of the total daily dose divided by the number of meals.
SEEN 2023 (26)	- If lifestyle changes (+/-medical-surgical therapy for weight loss) + non-insulin therapies do not achieve the goal of individualized control.	Start with basal insulin 10 U/day or 0.2-0.3 U/kg/day. Dose adjustment according to FCG (target < 110 mg/dl without hypoglycemia).
		Intensify with prandial insulin, preferably with rapid analogues and progressive adjustment (1st basal-plus -> 2nd bolus-basal)
SED 2018 (5)	- At the onset of the disease, if weight loss, severe ketonuria or cardinal symptoms especially with HbA1c >9%. -During follow-up, transient or permanent insulinization may be necessary in case of failure of non-insulin therapy.	Start with basal insulin 10 U/day or 0.2 U/kg/day and adjust according to FCG.
		Intensify with prandial insulin 4 U of rapid insulin analogue (0-10% of the basal dose) in the main intake or the one that generates the greatest postprandial hyperglycemia. Progressively add the 2nd or 3rd bolus according to evolution.

AACE, American Association of Clinical Endocrinologists; ADA, American Diabetes Association; EASD, European Association for the Study of Diabetes; FCG, fasting capillary glucose; HbA1c, glycated hemoglobin; SED, Spanish Diabetes Society; SEEN, Spanish Society of Endocrinology and Nutrition; U, Units.

In the document “Comprehensive Approach to People with T2DM” prepared by the Diabetes Knowledge Area of the Spanish Society of Endocrinology and Nutrition (SEEN), insulin use is recommended when the combination of lifestyle changes (including weight loss with medical-surgical therapy) and non-insulin therapies fails to achieve the individualized control goal (26). Likewise, as mentioned earlier, insulin will be the treatment of choice for patients with T2DM during pregnancy, in patients with intercurrent diseases, or in steroid treatment causing marked hyperglycemia, or in those in whom, due to circumstances (renal or hepatic insufficiency, adverse effects, etc.), the use of non-insulin drugs is contraindicated.

Once the decision to introduce insulin treatment has been made, basal insulin is the most suitable option. The decision to maintain other antidiabetic drugs should be assessed individually, considering that they may provide better control and reduce insulin requirements, which is associated with less weight gain. In the “Consensus on Insulin Treatment in T2DM” published by the Consensus and Clinical Guidelines Working Group of the Spanish Society of Diabetes (SED), it is recommended to continue treatment with metformin, dipeptidyl peptidase-4 inhibitors (DPP-4 inhibitors), GLP-1 RAs, and/or sodium-glucose cotransporter-2 inhibitors (SGLT-2 inhibitors) and consider stopping or reducing sulfonylurea/meglitinide treatment to decrease the risk of hypoglycemia and pioglitazone due to the increased risk of heart failure associated with this combination (5).

The main action of basal insulin is to reduce excessive hepatic glucose production and decrease overnight and between-meal hyperglycemia (3). Initial doses can be estimated based on body weight (0.1 to 0.2 U/kg/day) and the degree of hyperglycemia (for an HbA1c >8%, 0.2 to 0.3 U/kg/day may be considered), with individualized titration during the first few days or weeks (4). Special attention should be paid to the pharmacokinetic and pharmacodynamic profiles of available insulins, and the dose and timing should be adapted to each individual’s needs. Numerous insulin formulations are available, and therapy advances aim to better mimic physiological patterns (3). Scientific evidence indicates that the use of long-acting insulin analogs such as detemir, glargine U100-U300, or degludec is preferable to human insulins with protamine like neutral protamine Hagedorn (NPH) insulin due to their lower risk of hypoglycemia and greater flexibility in administration timing (3).

When using insulin in patients with T2DM, awareness of the potential risk of overt basalization is crucial. Clinical signs that may indicate overt basalization include a basal dose exceeding 0.5 U/kg/day, a significant difference (>50 mg/dL) between nighttime and basal glucose, the presence of hypoglycemia, and elevated glycemic variability. If a situation of overt basalization is identified, the patient should be reassessed to further individualize therapy (3).

Many people with T2DM may eventually need prandial insulin in addition to basal insulin to achieve glycemic goals. If the individual is not yet on treatment with a GLP-1 RAs, initiating it before prandial insulin should be considered to minimize the risks of hypoglycemia and weight gain associated with intensive insulin therapy (3). For those who ultimately require prandial insulin, it is a safe approach to initiate treatment with a dose of 4 units or 10% of the basal insulin amount at the most important meal, with higher carbohydrate content, or the one with the greatest glycemic excursion. The prandial insulin regimen should be subsequently adjusted according to individual needs.

For most patients receiving insulin, aiming for an HbA1c of 7% to 7.5% is recommended, but glycemic goals should be individualized. In addition to HbA1c, insulin titration requires the use of multiple glycemic parameters, including fasting glucose, pre-meal or 1.5-2-hour postprandial glucose, and, when available, continuous glucose monitoring (CGM) data including time in range (TIR), time below range (TBR), and glucose management indicator (GMI) (28). In general, fasting and pre-meal glucose targets should be <110 mg/dl without hypoglycemia, and postprandial glucose targets should be <180 mg/dl, although they should be individualized based on comorbidities and each person’s clinical status (27). If available, the use of CGM in people treated with insulin is recommended to optimize glycemic control and minimize hypoglycemia (28).

## Current barriers to initiating insulin therapy in type 2 diabetes

Insulin therapy remains the cornerstone of treatment for many individuals with T2DM. However, more recent therapies have emerged to circumvent some undesirable effects associated with its use. Consequently, insulin treatment is sometimes reserved for patients with a longer disease duration or when other non-insulin therapies prove unsuccessful. Moreover, the effectiveness of insulin treatment depends significantly on its appropriate use, the careful selection of patients, training in dose adjustment based on intake, activity, or weight, and proper dose titration to achieve acceptable and safe glucose levels (29). The primary advantage of insulin use lies in its effectiveness across a wide range of patients and various glycemic control targets (30).

Nevertheless, there are still various barriers to initiating insulin treatment in individuals with T2DM, which are detailed below (**Table 2**).

**Table 2:** Barriers to initiating insulin therapy.

Conditioning factor	Barrier	Strategy
**Patient**	- Adherence - Perception of failure	- Use of long-acting insulin - Dose recall systems - Diabetes education program
**Social environment**	- Social rejection	- Community education
**Treatment**	- Dosage - Hypoglycemia - Method of administration - Weight Gain	- Use of simple adjustment guidelines - Implementation of FGM/CGM systems - Documentation of episodes by the patient - Determination of a greater number of self-monitoring blood glucose. - Implementation of FGM/CGM systems - Education on signs and symptoms - Adjustments in physical exercise - Instruction in the guidelines for dealing with hypoglycemia. - Simple, intuitive devices adapted to different physical limitations. - Instruction in management technique. - Design of specific devices for patients with needle phobia. - Healthy eating education. - Prescription of physical exercise - Choice of type of insulin based on the reported weight evolution.
**Professional**	- Healthcare team experience - Clinical Guidelines of Scientific Societies	- Instruction and education of entire healthcare team. - Constant review of glycemic control goals - Proper device handling and management technique - Efficacy of insulin as an initial control tool in severe hyperglycemia. - Efficacy in certain diabetes profiles, such as diabetes secondary to the use of corticosteroids.

CGM, Continuous Glucose Monitoring; FGM, Flash Glucose Monitoring.

### Hypoglycemia

Hypoglycemia is likely the primary barrier to insulinization. Around 25% of individuals with T2DM, undergoing insulin treatment for more than 5 years, experience clinically significant hypoglycemic events (31). Recent studies have described a higher rate of cardiovascular events in T2DM patients with hypoglycemia (32). Additionally, in elderly patients or those with CKD, the presence of hypoglycemia may be associated with traumatic falls and cognitive impairments, leading to hospitalization (29).

One of the major limitations in addressing the problem is the scarcity of reported hypoglycemic episodes by patients, often due to a lack of awareness, insufficient self-monitoring, a failure to record events, and fear of failure (33). Therefore, it is crucial to emphasize patient education regarding hypoglycemia, enabling them to recognize potential signs and symptoms of hypoglycemic episodes and to always carry glucose supplements and rescue medication to appropriately address potential hypoglycemic episodes. Special attention is warranted for patients over 65 years old with T2DM undergoing insulin treatment. These patients have a higher risk of hypoglycemia (34), with additional limitations associated with polypharmacy, comorbidities, cognitive impairments, and increased costs (35, 36).

### Lack of adherence

Non-adherence to treatment in chronic diseases such as T2DM represents a significant limitation. According to the World Health Organization (WHO), non-adherence is estimated to be present in 50% of patients with T2DM (6). Moreover, lower adherence has been observed in patients undergoing insulin treatment compared to those using oral medications. Possible factors associated with non-adherence include certain sociodemographic characteristics, forgetfulness, lack of knowledge or diabetes education, fear of potential side effects, lack of confidence in the treatment, and economic considerations (6).

### Glucose monitoring

The need for proper glucose level monitoring for accurate insulin dose adjustments can present an additional barrier to initiating insulin therapy. In recent years, CGM has facilitated this task for those patients who use it regularly (37). However, the complexity of using these systems for the elderly population, patients with other comorbidities, or those with neurocognitive limitations, along with the lack of funding for T2DM in various settings, poses real limitations in achieving proper dose adjustments.

### Insulin administration

Insulin is typically administered through the subcutaneous route. Occasionally, in a hospital setting, it may be administered intravenously, and more rarely, intramuscularly. Proper administration technique is crucial for a satisfactory treatment response and to prevent potential adverse effects.

There are some factors associated with insulin administration that can influence its correct use (37):

- Daily administration of 1-4 insulin injections and the need to rotate the injection site.
- Correct administration technique, especially in patients with little subcutaneous tissue to prevent intramuscular injection.
- Fear or needle phobia.
- Forgetting to administer insulin or errors in the administered dose.
- Lack of glucose monitoring before insulin administration.
- Neglecting proper timing of meals.
- Inadequate adjustment of insulin dose for physical exercise.
- Patient’s physical limitations affecting the proper use of injection devices, such as mobility issues, visual impairments, arthritis, etc.

### Social rejection

From a social perspective, some individuals exhibit significant reluctance towards using insulin due to the stigma associated with its mode of administration. This needle-related stigma can impact participation in certain social and occupational activities (35, 38).

### Weight gain

Weight gain, documented in many studies involving patients on insulin treatment, is a limitation, especially for those who are overweight or obese. While initial weight gain is sometimes related to improved glycemic control and decreased glycosuria, particularly in patients with poor previous control (6, 38).

### Sense of therapeutic failure

Often, patients perceive the initiation of insulin therapy as a failure in effectively managing their diabetes. They may believe that insulin initiation will bring changes to their lifestyle and potentially result in a loss of autonomy (36). In a systematic review, the main psychosocial barriers to insulin initiation were identified as: [1] lack of awareness of the need for insulin treatment, seeking alternative ways for glycemic control without insulin; [2] negative consequences of insulin therapy on lifestyle; [3] perception of insulin treatment as something inaccessible, impractical, and unacceptable; and [4] anxiety associated with insulin treatment (39).

### Lack of healthcare team experience

The lack of experience among healthcare professionals in managing patients on insulin therapy can lead to therapeutic inertia in the initiation and subsequent adjustment of insulin doses, resulting in complications associated with poor glycemic control (35). In a retrospective cohort study, it was observed that more than 50% of individuals with T2DM maintained HbA1c levels around 8% in the 3-5 years prior to starting insulin treatment (39, 40). These findings underscore the importance of establishing individualized glycemic control goals from the outset and closely monitoring patients initiating insulin therapy. The Diabetes Attitudes, Wishes and Needs (DAWN) study emphasized the importance of adequate training for primary care physicians and nurses. These groups need to acquire greater knowledge of injection techniques, dose adjustment protocols by non-medical personnel, and ensure better use of available resources to support patients initiating insulin therapy (36).

### Therapeutic inertia

Therapeutic inertia, defined by the ADA as “a lack of timely adjustment to therapy when a patient’s treatment goals are not met”, is another widely described barrier to insulinization in patients with T2DM. Despite clear guidelines advocating for timely initiation of insulin therapy when glycemic targets are not achieved with oral antidiabetic agents, various factors contribute to physician hesitancy. These may include concerns regarding patient acceptance, fear of hypoglycemia, perceived complexity of insulin regimens, and time constraints during consultations (41).

### Recent clinical guidelines

Current clinical guidelines also represent a barrier to insulin use. In the recommendations of the most recent consensus statements for managing hyperglycemia in T2DM, the use of insulin is primarily reserved for individuals in whom other therapeutic strategies have failed. This current positioning of insulin could contribute to the negative perception associated with insulin treatment (36).

## Discussion

The development of insulin formulations has been constant in recent years, with notable advances such as insulin purification to reduce antibody production and allergic reactions, changes in basal insulins to delay absorption and cover 24-hour needs with a single injection, and adjustments in prandial insulins to accelerate absorption and better control postprandial hyperglycemia (42). Clinical guidelines suggest early use of GLP-1 RAs and SGLT-2 inhibitors, in combination with metformin, irrespective of the initial HbA1c, due to their cardiovascular benefits and preservation of renal function (3, 4). Despite improvements in treatment and glucose monitoring, only 55.8% in the United States (43) and 56% in Spain (44) achieve glycemic goals. Therefore, insulin will be necessary for many patients as complementary therapy to achieve and maintain glycemic targets.

This article has outlined various barriers to initiating insulin therapy, including the need for subcutaneous administration of one or more injections per day, weight gain, and various psychosocial factors complicating patients’ adoption of insulin therapy (45). These barriers, among other factors, contribute to therapeutic inertia in initiating and intensifying insulin treatment and to patients’ non-adherence to insulin therapy (46).

In this regard, the constant evolution of insulin administration tools over the past 100 years has represented a pivotal advancement in the endeavor to overcome these barriers. The use of insulin pens has changed the lives of millions of people who suffer from diabetes as they are safe, simple to use, convenient, efficient, and less painful than conventional vials and syringes (47).

Also, the recent development of weekly-administered insulins, by reducing injection frequency, could contribute to improved adherence to insulin therapy, patients’ quality of life, and better acceptance and satisfaction with treatment. For example, weekly-administered GLP-1 RAs have demonstrated greater efficacy, higher adherence, and greater treatment satisfaction compared to daily-administered agonists (48, 49).

**Figure 3:**
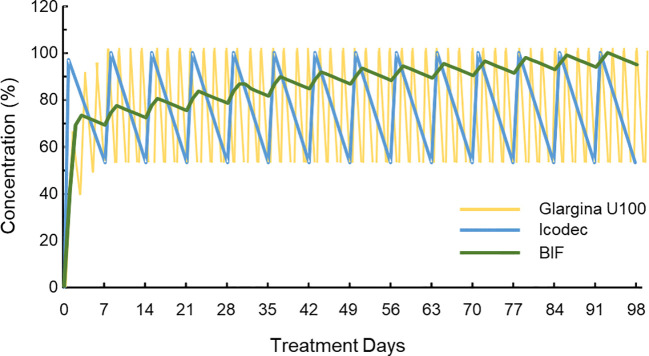
Simulated pharmacokinetic profiles of insulin glargine U100 (injected once daily), insulin icodec and basal insulin Fc BIF (both injected once weekly with a loading dose at first administration) Modified from (56). The pharmacokinetic profiles of one daily dose of insulin (glargine U100) and two weekly doses (icodec and BIF) are represented. The Y-axis represents the circulating concentration of each insulin expressed as a percentage, with 100% being the maximum concentration once steady state is reached. It can be observed that the increase in the half-life (glargine U100 the smaller, BIF the larger) means that it takes longer to reach the equilibrium state, although, once this is reached, the frequency of peak-to-valley oscillations is reduced and, in the case of BIF, the decrease in circulating levels is also significantly attenuated each time the trough is reached. BIF, basal insulin Fc.

Currently, there are two weekly-administered basal insulins in various stages of clinical development: icodec insulin and BIF basal insulin (insulin efsitora alfa; LY3209590). Icodec insulin is an insulin analog with three amino acid changes compared to human insulin and a 20C fatty acid chain attached to amino acid B29 through a hydrophilic bond. These changes facilitate strong binding to circulating albumin, reduced enzymatic degradation, and attenuated clearance after receptor binding (50). Icodec insulin has been evaluated in various phase 3 studies in patients with T2DM and type 1 diabetes, included in the ONWARDS program (51–56). BIF basal insulin is a fusion protein of an insulin chain with the extended-action IgG Fc fragment (IgG2Fc). This fusion allows for very slow clearance and weekly administration (57). Currently, this insulin has completed phase 2 of clinical development and is entering phase 3. The longer half-life of basal insulins is associated with lower peak-valley fluctuations and potentially lower risk of hypoglycemia, although with a longer time to reach a stable level after initiating or changing doses (58) (**Figure 3**). A recent meta-analysis of icodec insulin in patients with T2DM, including only the 3 phase 2 studies, showed a significant reduction in HbA1c, albeit of smaller magnitude (-0.20%; 95% CI: -0.33, -0.07%; *P*=0.002), with an increase in TIR (+6.6%), without a significant increase in hypoglycemia compared to glargine U100 insulin (59). However, the use of weekly insulins has not yet received regulatory approval, and there is a need for real-world studies to demonstrate whether their use facilitates insulinization, improves adherence, and enhances patients’ quality of life.

In summary, while insulin is necessary in T2DM and is an effective and safe therapy, there are still barriers to initiating insulin therapy. The future introduction of weekly-administered basal insulins that reduce injection frequency may help overcome some of these barriers by lessening therapeutic inertia and increasing treatment adherence. These new insulins, by enabling earlier introduction of insulin, could result in an earlier reduction in HbA1c without an increased risk of hypoglycemia and contribute to preventing the onset or progression of chronic diabetes complications.

## Author contributions

AS-P: Writing – original draft, Writing – review & editing. AE: Writing – original draft. AB: Writing – original draft. FA-B: Writing – original draft, Writing – review & editing.
